# Advances in endoscopic resection techniques of small gastric tumors originating from the muscularis propria

**DOI:** 10.3389/fonc.2022.1001112

**Published:** 2022-08-25

**Authors:** Suliman Khan, Xiaona Cui, Safyan Nasir, Shoaib Mohammad Rafiq, Bo Qin, Qian Bai

**Affiliations:** ^1^ Department of Cerebrovascular Diseases, the Second Affiliated hospital of Zhengzhou University, Zhengzhou, China; ^2^ Department of Anesthesiaology, the Second Affiliated hospital of Zhengzhou University, Zhengzhou, China; ^3^ Department of Medicine, Allied/District Headquarter Hospital Faisalabad, Faisalabad, Pakistan; ^4^ Department of Medicine, District Headquarter Hospital Gujranwala, Gujranwala, Pakistan; ^5^ Translational Medical Center, the First Affiliated Hospital of Zhengzhou University, Zhengzhou, China

**Keywords:** gastric tumors, gastrointestinal stromal tumors, gastrointestinal endoscopy, snare assisted endoscopic resection, endoscopic mucosal resection, endoscopic submucosal dissection

## Abstract

Gastrointestinal stromal tumors are common gastrointestinal tumors typically originating from the muscularis propria layer of the stomach. Small gastric stromal tumors are usually detected incidentally during routine endoscopic examination. Although they may have malignant potentially, controversies remain regarding the need for endoscopic resection of small gastric stromal tumors originating from the muscularis propria. According to the guidelines of the European Society of Medical Oncology, all gastrointestinal stromal tumors >2 cm in size should be resected with endoscopic surveillance recommended for tumors <2 cm. Endoscopic resection including endoscopic mucosal dissection (EMD), endoscopic submucosal dissection (ESD), submucosal tunneling endoscopic resection and snare assisted endoscopic resection. However, EMD and ESD procedures may be accompanied with serious complications including perforation, bleeding, and abdominal infection. Snare-assisted endoscopic resection is an alternative approach and has the advantages of a shorter procedure time and a low rate of perforation or bleeding. This study summarizes the safety and feasibility of a novel snare-assisted endoscopic resection technique and highlights the pros and cons of the different endoscopic approaches currently used for subepithelia small gastric tumors.

## Introduction

Small gastric stromal tumors are common, often asymptomatic and usually detected incidentally during upper gastrointestinal endoscopy (1–3). Widespread uses of digestive endoscopy and advances in endoscopic ultrasonography (EUS) have resulted in the diagnosis of small gastric submucosal tumors (4). These tumors are mostly gastrointestinal stromal tumors which have malignant potential. Minimally invasive endoscopic resection has become increasingly popular as a method for removal of small submucosal tumors.

According to the National Comprehensive Cancer Network (NCCN) guidelines, tumor resection is the first-line treatment for non-metastatic gastrointestinal stromal tumors. Larger gastrointestinal stromal tumors (>5 cm) often have more aggressive morphologic features and proliferation activity such that laparoscopic resection or surgery is recommended for complete resection (5, 6). However, it is still controversial for the treatment criteria of gastrointestinal stromal tumors (<2 cm) because of their uncertain malignant potential. Periodical surveillance and follow-up are recommended for tumors ≤2 cm, since they are considered of very low risk of malignancy and metastasis (7). However, several researchers have emphasized that small tumors have intermediate or high risk and should be resected to confirm the diagnosis and to avoid further risk of malignancy (8, 9).

Currently, the main endoscopic approaches for small gastrointestinal tumors include endoscopic mucosal dissection (EMD), endoscopic submucosal dissection (ESD), submucosal tunneling endoscopic resection, and snare assisted endoscopic resection (10–12). EMD is not ideal as it cannot reliably ensure complete resection for relatively larger tumors. ESD reliably provides complete resection and an accurate pathological diagnosis but is associated with a risk of perforation or bleeding and requires a relatively long procedure time (13, 14). The alternative is a snare-assisted endoscopic resection procedure which, compared to ESD, include a shorter procedure time, a low rate of perforation or bleeding, and increased cost effectiveness (15). This study summarizes the safety, feasibility and utility of the novel snare-assisted endoscopic resection technique for small gastric tumors originating from the muscularis propria compared to the well-established techniques of EMR and ESD.

## Snare assisted endoscopic resection-accessories and technique

### Instruments and accessories

A single-channel flexible endoscope such as the EVIS GIF-Q260J, Olympus, Tokyo, Japan is used to perform the procedure. Other equipment included a snare (Boston Scientific, Ref; M00562650), a transparent cap (Olympus, Model No: D-201-11804), endoscopic clips (ROCC-D-26-195, Micro-Tech (Nanjing) Co., Ltd, Chinaor HX-610-135, Olympus), and a virtual input & output (VIO) electrosurgical generator (ICC 200 EA INT; ERBE, Tübingen, Germany). The endoscope and all accessories are sterilized by soaking in paracetic acid prior to use.

### Description of technique

**Figure 1:**
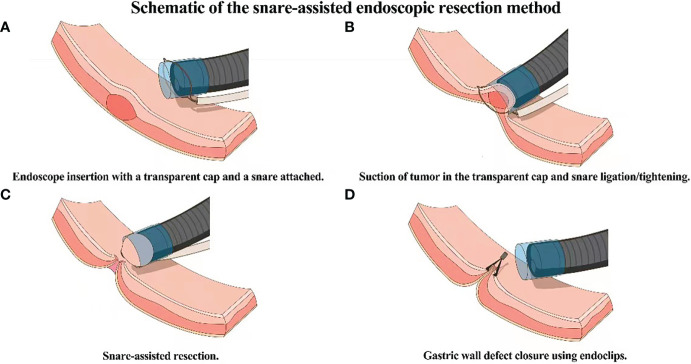
Schematic of the snare-assisted endoscopic resection method. **(A)** Endoscope entery into the stomach with a transparent cap and snare attached on the tip. **(B)** Suction of the tumor into the transparent cap and snare ligation. **(C)** Resection of the tumor. **(D)** Closure of the wound using endoclips.

Routine bowel preprations are performed before the procedure. After stomach cleansing using a saline solution, a single channel flexible endoscope with a transparent cap and a snare attached on the tip of the endoscope is introduced into the stomach. The lesion is observed and sucked into the transparent cap after which the snare is used to ligate the base of the tumor. The wire of the snare is tightened and a high frequency (35W) current is applied to resect the lesion. After the lesion is removed, any bleeding is controlled with electro coagulation forceps. Endoclips are then used to close the wound site and the resected specimen is sent for histopathologic examination (**Figure 1**).

## Snare assisted endoscopic resection- indications and limitations

Controversies remain concerning the specific indications for endoscopic resection for small gastric tumors originating from muscularis propria. Based on current experience, patients with intraluminal submucosal tumors without ulceration, and maximal tumor diameter of 2 cm measured by endoscopic ultrasonography (EUS) or computed tomography (CT), and no evidence of lymph node involvement or distant metastasis are candidates for snare-assisted endoscopic resection (16, 17). Differentiation between potentially malignant gastrointestinal stromal tumors and other benign or non-neoplastic lesions is extremely difficult using image methods, especially for small lesions. In addition, some studies have reported that small gastric stromal tumors (<2 cm) may be of intermediate or high risk. Pang et al. reported a series with tumor size <2 cm in which 9 patients (3.9%) were in the intermediate risk group and 2 patients (0.9%) were in the high risk group (18). Yang et al. reported a series in which 7 cases (2.5%) were of intermediate risk and 10 cases (3.6%) and high risk in gastric stromal tumors <2 cm (19). Gao et al. reported that EUS-suspected gastrointestinal stromal tumors larger than 9.5 mm may be associated with significant progression (20).

In our center 62 patients (21 men and 41 women) with small gastric submucosal tumors were managed with a snare-assisted endoscopic resection. The mean age was 53.7 ± 11 years (range 28-73 years old). The mean tumor size was 0.64 ± 0.16 cm (median 0.60, range 0.31-0.97 cm). The technical success rate was 100%. The mean procedure time was 19.8 ± 9 minutes (median 20, range 9-30 min). There were no complications during or after the procedure. Due the small amount of mucosal damage wound clips were not required in 23 (37%) patients. Twenty-five (40.3%) of the tumors were located in the fundus near the cardia, 32 (51.6%) were located in the body and 5 (8%) were in the antrum. During 3-22 months of follow up, no recurrence or metastasis have occurred.

The snare-assisted endoscopic resection methods have several limitations. First, this method might not be suitable for gastric tumors located in the gastric cardia. When dissecting tumors located in the gastric cardia, the small size of the gastric cardia limits the performance of this method. Second, the direction of the traction can be provided on the oral side only. However, in most cases, this traction direction is sufficient for resection of tumors located in the fundus and corpus. Third, perforation cannot be completely avoided in all cases. Dissection of gastric tumors with a wide base will cause a wide defect in the stomach wall and likely result in a greater perforation rate. However, further investigation is needed to explore the risk factors associated with the perforation rate when using this method.

## Other endoscopic approaches- techniques, safety and associated complications

Endoscopic methods other than snare-assisted endoscopic resection include, EMD, ESD, endoscopic submucosal excavation, submucosal tunneling endoscopic resection, and endoscopic full-thickness resection (21–23). All the procedures are performed under anesthesia with tracheal intubation.

Endoscopic Submucosal Dissection Technique: The endoscopic submucosal dissection mainly includes three steps: (i) identifying and marking the tumor boundaries; (ii) injecting saline mixed with indigo carmine into the submucosal layer; (iii) incising the mucosal layer with hook knife and dissecting the tumor using an IT knife.

Endoscopic Mucosal Dissection Technique: The endoscopic mucosal Resection technique is an improvement in the ESD procedure. The procedure includes. (i) Marking the tumor boundaries and injecting the solution into the submucosal layer; (ii) Incising the mucosal layer longitudinally; (iii) Dissecting the submucosal and muscular tissue to expose and remove the tumor completely; (iv) Checking the wound and clipping the incision.

EMD or ESD can be used successfully to treat gastric stromal tumors originating from the superficial muscularis propria layer, but when the tumor originates from the deep muscularis propria layer or has a tight connection with the underlying muscularis propria layer or serosal layer, the complete resection rate decreases and the rate of complications increases (24). If a small gastrointestinal stromal tumor is deemed to have a high potential for malignancy and postoperative recurrence, surgical resection is the best option to achieve sufficiently deep and lateral margins (25).

In a large-scale study of ESD treatment of 144 patients with muscularis propria tumors, the complete resection rate was 92.4% and no recurrence was detected. Several studies have demonstrated that endoscopic full-thickness resection can be the diagnostic and definitive treatment for these tumors, with a success rate of 90% and low incidence of complications (26, 27). Therefore, for tumors <2 cm, besides periodic follow-up using EUS, endoscopic resection may be a good option. Endoscopic full-thickness resection allows en bloc resection by resecting the tumor and the surrounding serosal layer, but primary closure may be difficult, and perforation, serious bleeding and peritonitis may occur, which contraindicates its use for small muscularis tumors (28).

Endoscopic resection of muscularis propria tumors in different locations of the gastrointestinal tract can encounter difficulties; therefore, these tumors should be treated *via* different endoscopic approaches. For example, the complications rate for ESD/EFTR varies among the different locations of the stomach with the fundus being regarded as a difficult area for endoscopic resection (29). Perforation associated with endoscopic treatment is a serious complication that could confer an increase rate of mortality and major morbidity. Perioperative bleeding is another major complication of endoscopic resection of upper gastrointestinal tumors. The incidence of perioperative bleeding reported by recent studies ranges from 0 to 38.7%, which shows a high degree of variability (30, 31).

## Conclusion and future direction

Advancement in endoscopic resection techniques including development of EMD and ESD has resulted in increased use of endoscopy for treatment of small gastric submucosal tumors. The selection of a particular technique depends on the size and location of the tumor. Overall, numerous studies have confirmed that these approaches are effective in terms of safety, and outcomes. Although both EMD and ESD are effective and are generally preferred for larger and deep lesions an approach that reduces procedure time, costs, and reduces hospital stay would be preferred. The snare-assisted method for the removal of gastric submucosal tumors smaller than 2 cm has the advantages of short procedure time, rare complications, short hospital stay and rapid post-procedural recovery. This is a simple and effective method for the treatment of small gastric submucosal tumors and achieves clinical success rates equal to or better than achieved with other endoscopic resection techniques (e.g., EMR and ESD) with advantages of efficacy, quickly and cost effectiveness.

The management of small gastric stromal tumors, particularly originating from the muscularis propria of the stoamch, is evolving towards an ultra minimally invasive approach. Where available, snare-assisted endoscopic resection can reduce procedure time, hospital stay, and cost of small gastric stromal tumors management. Specific treatment algorithms for snare assisted endoscopic resection, as are established for EMR and ESD, are needed. There is also a need for multinational collaboration and a consensus on training and credentialing pathway for snare assisted endoscopic resection technqiue, and on areas of future research necessary for widespread adoption.

## Data availability statement

Publicly available datasets were analyzed in this study. This data can be found here: The data can be provided on request to the corresponding author. (Qian Bai, email: baiqian@zzu.edu.cn.)

## Ethics statement

The studies involving human participants were reviewed and approved by the ethics committe of the Second Affiliated Hospital of Zhengzhou University. The patients/participants provided their written informed consent to participate in this study.

## Author contributions

Study concept and design: SK, QB, BQ. Manuscript writing: SK, SN, SR. Analysis and interpretation of data: XC, SN. SR. Acquisition of data: XC, SN. Critical revision of manuscript and video: SK, QB. Funding obtained: SK, QB. All authors contributed to the article and approved the submitted version.

## Funding

This study was financially supported by grants from Henan province innovation talents of science and technology plan (No. SB201901045) and hepatobiliary foundation of Henan Charity General Federation (No: GDXZ2019006).

## Conflict of interest

The authors declare that the research was conducted in the absence of any commercial or financial relationships that could be construed as a potential conflict of interest.

## Publisher’s note

All claims expressed in this article are solely those of the authors and do not necessarily represent those of their affiliated organizations, or those of the publisher, the editors and the reviewers. Any product that may be evaluated in this article, or claim that may be made by its manufacturer, is not guaranteed or endorsed by the publisher.
